# To live and die well with neurocognitive disorders

**DOI:** 10.1002/alz70858_103696

**Published:** 2025-12-26

**Authors:** Felix Pageau, Sonia Bélanger, Jeane Day, Marie Christine Le Bourdais, Paolo Vitali, Ziad Nasreddine, Guy Lacombe, Thomas Tannou

**Affiliations:** ^1^ Université Laval, Québec, QC, Canada; ^2^ Ministre des Ainés ‐ Ministère de la Santé et des Services sociaux, Quebec, QC, Canada; ^3^ Alzheimer Society of Montreal, Montreal, QC, Canada; ^4^ The McGill University Research Centre for Studies in Aging, Montreal, QC, Canada; ^5^ MoCA Clinic and Institute, Greenfield Park, QC, Canada; ^6^ CIUSSS de l'Estrie‐CHUS, Sherbrooke, QC, Canada; ^7^ CRIUGM, CIUSS Centre‐Sud de l'Ile de Montréal, Montreal, QC, Canada

## Abstract

The end of life in Major Neurocognitive Disorder (MND) is often shaped by a range of medical complications. These can include aspiration pneumonia, hip fractures, subdural hemorrhages, untreated cancer, or pyelonephritis. Regardless of the cause, the provision of appropriate, compassionate care remains a critical element. In Quebec, the recent legalization of medical assistance in dying (MAiD) through advance requests for individuals with MND introduced new dimensions to how these individuals approach death. What are the ethical implications of this change? In this presentation, we aim to explore how death occurs from MND, specifically in the context of Quebec in 2025, and the ethical dilemmas associated with this shift.

To understand these ethical concerns, it is also crucial to examine what constitutes a “good death” in MND. A “good death” is not only about the medical management of end‐of‐life symptoms but also about the opportunity to maintain dignity, receive emotional support, and, for some, exercise personal autonomy in decision‐making. We will discuss these concepts and present an article we have written and published in the scientific journal *Mortality*. This article discusses the difficult choices faced by individuals with MND: whether to die quickly and painlessly or to take time to say goodbye to loved ones—a decision that is often fraught with emotional and ethical challenges.

These issues were raised during semi‐structured interviews conducted by Dr. Pageau, who gathered data as part of a research project on the determination of care levels in geriatric patients in Quebec. The interviews provided valuable insights into the personal, social, and ethical dimensions of end‐of‐life decision‐making for people with MND.

We will present the results of this study to help foster an ethical discussion about the complex issues surrounding care and the dying process for individuals with neurocognitive disorders.

